# Direct cost of maintenance of totally implanted central venous catheter patency

**DOI:** 10.1590/1518-8345.2263.3004

**Published:** 2018-07-16

**Authors:** Rafael Fernandes Bel Homo, Antônio Fernandes Costa Lima

**Affiliations:** 1 MSc, RN, Clinical Research, Instituto do Câncer Do Estado de São Paulo, São Paulo, SP, Brazil.; 2 PhD, Associated Professor, Nursing School, Universidade de São Paulo, São Paulo, SP, Brazil.

**Keywords:** Oncology Nursing, Drug Therapy, Catheters, Care Nursing, Costs and Cost Analysis, Cost Control

## Abstract

**Objective:**

to identify the average direct cost of maintaining the patency of totally implanted central venous catheter with heparin at a Day Hospital of a public hospital of high complexity specialized in the treatment of cancer patients, and estimate the average direct cost of replacing heparin with sodium chloride 0.9%.

**Method:**

quantitative, exploratory-descriptive study, with a sample of 200 non-participant observations of the maintenance of totally implanted central venous catheters with heparin. The average direct cost was calculated by multiplying the (clocked) time spent by professionals to complete the procedure by the direct unit cost of workforce, added to the cost of materials and solutions.

**Results:**

the estimated total direct cost of catheter maintenance with heparin was US$ 9.71 (SD=1.35) on average, ranging from US$ 7.98 to US$ 23.28. The estimated total direct cost of maintenance with 0.9% sodium chloride in the place of heparin was US$ 8.81 (SD=1.29) on average, resulting in a reduction of US$ 0.90 per procedure.

**Conclusion:**

the results contributed to propose strategies to assist in cost containment/minimization in this procedure. The replacement of heparin by 0.9% sodium chloride proved to be an option to reduce the total average direct cost.

## Introduction

Oncological treatment requires periodic actions to maintain the patency of totally implanted central venous catheter (CVC-TI), a safe and effective device for intravenous chemotherapy, and to maintain the integrity of the venous network of the patient^1^ ^-^ ^2^. This procedure can be performed with heparin or 0.9% sodium chloride^1^ ^-^ ^2^, and requires qualified human resources and specific materials whose costs are unknown by health organizations and the professionals responsible for carrying out the process. Such lack of knowledge may compromise the decision-making process when it comes to efficient resource allocation, in the sense of minimizing costs without impairing the quality of the assistance provided.

Increasing costs have called the attention of hospital managers, health professionals and payers of care, especially in hospital areas with considerable expenses and at the same time scarce financial resources, which increases the emphasis on cost control^3^. 

Nursing professionals represent a significant part of the staff in health organizations and it is up to them to make decisions regarding structure, processes, human resources and institutional outcomes. Therefore, knowledge of the costs associated with the provision of nursing services may support their qualified participation in redefining priorities, rationalizing limited resources and monitoring productivity^4^.

Considering that nurses have been responsible for the coordination of health care teams, units/sectors/services or even material management, it is imperative that they acquire knowledge regarding health costs^5^.

In view of the relevance of the procedure discussed here, in order to ensure the feasibility of chemotherapy treatment to cancer patients, it is worth noting that knowledge on incurred costs may help in the decision-making process, allowing cost control. The objective of this study was to identify the average direct cost of maintenance of CVC-FI patency with heparin at the Day Hospital of a highly complex public hospital specialized in the treatment of cancer patients and to estimate the average direct cost of replacement of heparin by 0.9% sodium chloride for maintaining CVC-TI patency. 

## Method

This quantitative, exploratory-descriptive study was conducted at the Day Hospital of the Octávio Frias de Oliveira Cancer Institute of the State of São Paulo, after approval by the Ethics and Research Committees of the proposing (Opinion n°: 1,508,586) and co-participant (Opinion n°: 1,517,226) institutions. 

The Day Hospital, located at the 12th floor of the institution, is intended for the care of patients who need blood transfusion, administration of antibiotics, intrathecal chemotherapy, Bacillus Calmette-Guérin (BCG) therapy, hydration, growth factor, erythropoietin, preparation for outpatient surgeries, and introduction and maintenance of CVC-TI patency. Its physical structure has seven treatment rooms, comprising 35 leasing positions (27 armchairs and eight stretchers), a room for patients in respiratory isolation, an emergency room, office, three bathrooms, a washing room, a dining room, and a medical comfort room.

The nursing team of the Day Hospital is composed of 20 professionals, seven nurses and 13 nursing technicians, who work in morning shifts of 12x36 hrs. The team on duty A has three nurses and seven technicians and the team on duty B has three nurses and six technicians. A nurse fulfills a work day of 8 hours and works in both shifts, but all professionals have a contract workload of 206 hours/month.

CVC-TI patency maintenance, also called CVC-TI heparinization, occurs daily in the Day Hospital. Patients are previously scheduled to attend the institution, with prediction of returning every 28 days. The procedure follows the technique recommended in the current institutional protocol for the use of heparin.

After the user arrives at the Day Hospital, the nurse asks for the materials and solutions that will be used, and he or a nursing technician collects the material at the Material and Drug Assistance Center (MAC) located on the 11th floor. Subsequently, the nurse prepares the material and the medication, starting with asepsis of the area where the CVC-TI is implanted, punctures the catheter with a Huber needle, performs the heparinization, removes the Huber needle and discards the materials used.

The sample size, calculated based on a confidence level of 95% and a tolerable statistical error of 10%, corresponded to at least 100 non-participant observations of CVC-TI heparinizations. Data collection occurred in the period from July to August 2016, totaling 200 observations.

The procedure was divided into four steps: 1st) collection of material in the MAC at the 11th floor; 2nd) material preparation and asepsis, which could be performed by a nurse or a nursing technician; 3rd) puncture and CVC-TI heparinization (exclusively performed by a nurse); and 4th) disposal of the material, which could be performed by a nurse or a technician.

The direct costs of CVC-TI heparinization were estimated. Direct cost is defined as the monetary expenditure that is incurred by the production of a product or provision of a service in which there is the possibility of identification with the product or department. It refers to the costs that can be identified and quantified^6^, mainly represented by costs with direct workforce, materials, drugs, solutions and equipment used in the care process.

Direct workforce comprises the personnel who work directly on a product or service provided, whose work time can be measured and identity at individual level can be known. Costs involve salaries, social charges, holiday provisions, and 13th salary^6^.

The cost of the direct nursing professional workforce was calculated based on the average salaries (salary, benefits, gratifications, and social charges) per category (nurse and technician) informed by the Financial, Planning and Control Department. The Purchasing Sector of the Hospital provided information on the costs of purchasing materials and solutions.

As the execution of a procedure may require the consumption of different quantities of supplies, it is possible to establish the average direct cost $\left[\;\bar{C\left(P_{i}\right)}\;\right]$ from the average direct cost of the set of materials $\left[\;\bar{C{\left(P_{i}\right)}_{mat}}\;\right]\;$, solutions $\left[\;\bar{C{\left(P_{i}\right)}_{sol}}\;\right]\;$ and workforce $\left[\;\bar{C{\left(P_{i}\right)}_{mob}}\;\right]$ ^7^: $\bar{C\left(P_{i}\right)}=\bar{C{\left(P_{i}\right)}_{mat}}+\bar{C{\left(P_{i}\right)}_{sol}}+\bar{C{\left(P_{i}\right)}_{mob}}$ (equation 1).

The $\bar{C{\left(P_{i}\right)}_{mat}}$ is obtained by summing the average costs $\left[\bar{Cm_{k}\;}\right]$ of each material [k] used in procedure^7^: $\bar{\;C{\left(P_{i}\right)}_{mat}}=\overset{n}{\underset{k=1}{\sum }}\bar{Cm_{k}}$ (equation 2). 

Thus, we obtained the $\bar{C{\left(P_{i}\right)}_{mat}}$ of each material by multiplying the average quantity used $\left[\bar{qm_{k}}\right]$ by its average unit price $\bar{Pmu_{k}}$: $\bar{Cm_{k}}=\bar{qm_{k}}\cdot \bar{Pmu_{k}}$ (equation 3).

By replacing equation (3) in the equation (2), the following equation was obtained: $\bar{\;C{\left(P_{i}\right)}_{mat}}=\overset{n}{\underset{k=1}{\sum }}\;\left(\;\bar{q_{mk}}\;\cdot \bar{Pmu_{k}}\right)$ (equation 4).

The $\bar{C{\left(P_{i}\right)}_{sol}}$ is obtained by summing the average costs $\left[\bar{Cs_{k}\;}\right]$ of each solution/drug consumed^7^: $\bar{\;C{\left(P_{i}\right)}_{sol}}=\overset{n}{\underset{k=1}{\sum }}\bar{Cs_{k}}$ (equation 5).

Therefore, the $\bar{C{\left(P_{i}\right)}_{sol}}$ was obtained by multiplying the average quantity of the solution/drug $\left[\bar{qs_{k}}\right]$ by its mean unit price $\left[\bar{Psu_{k}}\right]$: $\bar{Cs_{k}}=\bar{qs_{k}}\cdot \bar{Psu_{k}}$ (equation 6).

By replacing equation (6) in the equation (5), a more detailed equation was obtained: $\bar{\;C{\left(P_{i}\right)}_{sol}}=\overset{n}{\underset{k=1}{\sum }}\;\left(\;\bar{qs_{k}}\;\cdot \bar{Psu_{k}}\right)$ (equation 7).

The $\bar{C{\left(P_{i}\right)}_{mob}}$ is obtained by the sum of the average costs of each professional category (nurses and nursing technicians) $\left[\bar{Ch_{c}\;}\right]$ participating in the procedure^7^ $\bar{\;C{\left(P_{i}\right)}_{mob}}=\overset{n}{\underset{c=1}{\sum }}\bar{Ch_{c}}$ (equation 8).

The $\bar{\;C{\left(P_{i}\right)}_{mob}}$ was obtained by multiplying the average time spent by each category [*c*] in the procedure $\left[\bar{t_{c}\;}\right]$ by the average unit cost of the workforce $\left[\bar{Su_{c}\;}\right]$: $\bar{Ch_{c}}=\bar{t_{c}}\cdot \bar{Su_{c}}$ (equation 9).

By replacing equation (9) in the equation (8), a more detailed equation was obtained: $\bar{\;C{\left(P_{i}\right)}_{mob}}=\overset{n}{\underset{c=1}{\sum }}\;\left(\;\bar{t_{c}}\;\cdot \bar{Su_{c}}\right)$ (equation 10).

Finally, by replacing equations (4), (7) and (10) in the equation (1), we obtain equation (11)^7^: $\bar{C\left(P_{i}\right)}=\overset{n}{\underset{k=1}{\sum }}\;\left(\;\bar{q_{k}}\;\cdot \bar{Pu_{k}}\right)+\overset{n}{\underset{k=1}{\sum }}\;\left(\;\bar{qs_{k}}\;\cdot \bar{Psu_{k}}\right)+\overset{n}{\underset{c=1}{\sum }}\;\left(\;\bar{t_{c}}\;\cdot \bar{Su_{c}}\right)$ (11). Then, for calculation of the $\bar{C\left(P_{i}\right)}$, the following intervenient variables were defined: average quantity of materials $\left[\bar{qm_{k}\;}\right]$; average unit price of each material $\left[\bar{Pmu_{k}\;}\right]$; average quantity of solutions/drugs $\left[\bar{qs_{k}\;}\right]$; average unit price of each solution/drug $\left[\bar{Psu_{k}\;}\right]$; average time spent per professional category $\left[\bar{t_{c}\;}\right]$; average unit salary mass of each professional category $\left[\bar{Su_{c}\;}\right]$ ^7^.

For calculations, the Brazilian currency (R$) was originally used and converted into US$ using the current exchange rate of US$ 0.31/R$, quoted in 07/29/2016, provided by the Central Bank of Brazil.

## Results

During the period of data collection, all nursing professionals at the Day Hospital, from shifts A and B, who performed 200 CVC-TI heparinizations were observed.

Among the 200 patients who composed the sample, the majority (70%) were female, aged between 18 and 102 years, with a mean age of 51.2 years (SD=16.7), and the most prevalent types of cancer was Malignant neoplasms of lymphoid, hematopoietic and related tissues (C81-C96) (35.0%); Malignant neoplasms of the breast (C50) (27.5%); and Malignant neoplasms of the digestive organs (C15-C26) (20.0%), according to the International Classification of Diseases (ICD-10). Regarding the site of insertion of the CVC-TI, 176 (88%) catheters were implanted in the right hemithorax; 21 (10.5%) in the left hemithorax; and three (1.5%) in the right femoral vein.

The first step of the procedure, “collection of material in the MAC at the 11th floor”, was performed by nurses or nursing technicians and the second, third and fourth steps, “preparation of material and asepsis”, “puncture and CVC-TI heparinization” and “disposal of material”, only by nurses.

The time spent in the first step varied from four to 19 minutes, with an average of 8.82 (SD=2.50), median and mode of eight minutes. Table 1 shows that this step, whose average total direct cost with the nursing team corresponded to US$ 0.92 (SD=0.32), was mostly performed (96%) by nursing technicians.

The second step, “preparation of material and asepsis”, lasted from two to 13 minutes, with an average of 6.39 (SD=2.13), median of six and mode of seven minutes. Table 2 shows that the cost with material (US$ 5.55 - SD=0.14) was the main value contributing to the average total direct cost of this step, followed by cost with nurses (US$ 1.29 - SD=0.46). The only nonstandard material used in the procedure, named additional material, was valve connectors (US$ 0.71/unit - 25 units/US$ 17.75).

As for the material consumed, Huber needles, which had the highest unit cost (US$ 4.15), represented the item with the highest impact in the cost (200 units - US$ 830.00), followed by sterile gloves (US$ 0.22/pair - 378 pairs/US$ 83.16), disposable sterile field (US$ 0.36/unit - 200 units/US$ 72.00), and gauze (US$ 0.09/package - 380 packages/US$ 34.20).

Heparin was the item with the highest unit cost (US$ 0.71) and the most important in the total cost with solutions, totaling US$ 143.42 due to the consumption of 202 ampoules, followed by 0.5% alcohol chlorhexidine (US$ 0.14/unit - US$ 28.00/200 units) and 0.9% sodium chloride (US$ 0.05/ampoule - US$ 20.30/406 ampoules).

**Table 1:** Distribution of the observations related to the first step “collection of material in the MAC* at the 11th floor”, according to cost with personnel. São Paulo, SP, Brazil, 2017

Observations	n	Mean US$^†^	SD^‡^ US$^†^	Median US$^†^	Mode US$^†^	Minimum-Maximum US$^†^
Cost with Nurses	08	1.75	0.55	1.60	1.60	1.2-3.0
Cost with Technicians	192	0.88	0.25	0.80	0.80	0.40-1.90
Total average direct cost with personnel	200	0.92	0.32	0.80	0.80	0.40-3.00

*MAC - Material and Drug Assistance Center; †Conversion rate: US$ 0.31/R$, quoted in 29/07/2016, provided by the Central Bank of Brazil; ‡SD - Standard deviation.

**Table 2:** Distribution of the observations related to the second step “preparation of material and asepsis” according to cost with personnel, material, solutions and additional material. São Paulo, SP, Brazil, 2017

Observations	n	Mean US$^*^	SD^†^ US$*	Median US$^*^	Mode US$^*^	Minimum-Maximum US$^*^
Cost with Nurses	200	1.29	0.46	1.20	1.40	0.40-4.00
Cost with Material	200	5.55	0.14	5.57	5.51	5.20-5.84
Cost with Solutions	200	0.96	0.07	0.96	0.96	0.95-1.71
Cost with Additional Material	25	0.71	0.00	0.71	0.71	0.71-0.71
Total average direct cost	200	7.89	0.67	7.86	7.46	6.55-11.41

*Conversion rate: US$ 0.31/R$, quoted in 29/07/2016, provided by the Central Bank of Brazil; †SD - Standard deviation

**Table 3:** Distribution of the observations related to the second step “puncture and CVC-TI heparinization”* according to cost with personnel, material and solutions. São Paulo, SP, Brazil, 2017

Observations	n	Mean US$^†^	SD^‡^ US$^†^	Median US$^†^	Mode US$^†^	Minimum-Maximum US$^†^
Cost with Nurses	200	0.49	0.54	0.40	0.40	0.20-7.20
Cost with Material	03	4.76	0.73	4.42	-	4.27-5.60
Cost with Solutions	03	0.33	0.53	0.05	-	0.01-0.95
Total average direct cost	200	0.56	1.08	0.40	0.40	0.20-13.75

*CVC-TI - Totally implanted central venous catheter; †Conversion rate: US$ 0.31/R$, quoted in 29/07/2016, provided by the Central Bank of Brazil; ‡SD - Standard deviation

The time spent in the third step, “puncture and CVC-TI heparinization”, varied from one to 18 minutes, with an average of 2.33 (SD=1.70), median and mode of two minutes. The maximum time was spent in a single procedure that required a new puncture for insertion of the CVC-TI, which took further time for the nurse to check the materials and reevaluate the puncture site. According to Table 3, the cost with nurses (US$ 0.49 - SD=0.54) was the main contributor to the total average direct cost.

The cost with material and solutions in the third step of the procedure was related to three observations of occasions when there was some difficulty that required a new puncture or when a complication occurred, as for example, absence of blood reflux. The costs with consumption of Huber needles (3 units - US$ 12.45), gauze (5 packages - US$ 0.45) and disposable sterile field (1 unit - US$ 0.37) stood out; as for the solutions. There were costs with heparin (1 ampoule - US$ 0.71) and 0.9% sodium chloride (3 ampoules - US$ 0.15).

The fourth step, “disposal of material”, had a duration varying from one to four minutes, with a mean of 1.52 (SD=0.66), median and mode of one minute. It is observed in the Table 4 that the cost with nurses (US$ 0.31 - SD=0.13) prevailed as contributor for the composition of the ADC (US$ 0.34 - SD=0.14).

The average total duration of the procedure was 19.06 minutes (SD=3.84), ranging from 12 to 36 minutes, with median of 19 and mode of 20 minutes. The average total direct cost of the four steps involved in the heparinization of the CVC-TI and the estimated use of sodium chloride 0.9% (salinization) is shown in Table 5.

**Table 4:** Distribution of the observations related to the fourth step “disposal of material” according to cost with personnel, material and solutions. São Paulo, SP, Brazil, 2017

Observations	n	Mean US$*	SD^†^ US$*	Median US$*	Mode US$*	Minimum-Maximum US$*
Cost with Nurses	200	0.31	0.13	0.20	0.20	0.20-0.80
Cost with Material	196	0.03	0.02	0.02	0.02	0.01-0.11
Cost with Solutions	200	0.01	0.00	0.01	0.01	0.01-0.01
Total average direct cost	200	0.34	0.14	0.27	0.23	0.21-0.92

* Conversion rate: US$ 0.31/R$, quoted in 29/07/2016, provided by the Central Bank of Brazil; †SD - Standard deviation

**Table 5:** Distribution of the average total direct cost in the four steps of heparinization of CVC-TI* and of the estimated average total direct cost with the use of 0.9% sodium chloride. São Paulo, SP, Brazil, 2017

Maintenance of permeability with heparin (heparinization)	Total average direct cost US$^†^	SD^‡^ US$^†^	Minimum-Maximum US$^†^
Step 1	0.92	0.32	0.40-3.00
Step 2	7.89	0.67	6.55-11.41
Step 3	0.57	1.17	0.20-13.75
Step 4	0.34	0.14	0.21-0.92
Total average direct cost of heparinization	9.71	1.35	7.98-23.28
Maintenance of permeability with sodium chloride 0.9% (salinization)	Estimated total average direct cost US$^†^	SD^‡^ US$^†^	Minimum-Maximum US$^†^
Step 1	0.92	0.32	0.40-3.00
Step 2	6.99	0.65	5.67-9.82
Step 3	0.56	1.02	0.20-12.87
Step 4	0.34	0.14	0.21-0.92
Total average direct cost of salinization	8.81	1.29	7.10-21.52

*CVC-TI - Totally implanted central venous catheter; †Conversion rate: US$ 0.31/R$ quoted in 29/07/2016, provided by the Central Bank of Brazil; ‡SD - Standard deviation

It should be emphasized that, for salinization of the CVC-TI, no additional material had to be added according to the current procedure at the Day Hospital and it would still be possible to remove the 20ml syringe and a 0.9% sodium chloride ampoule, as well as the heparin ampoule.

Comparing the total ADC of maintenance of CVC-TI permeability with heparin (US$ 9.71 - SD=1.35) and with 0.9% sodium chloride (US$ 8.81), it was evident that the latter promoted a reduction of US$ 0.90 per procedure. Considering the average of 280.75 procedures per month performed in the HD and extrapolating the total ADC obtained for heparinization, this cost would correspond to US$ 2,726.08/month and US$ 32,712.99/year; and for salinization, US$ 2,473.41/month and US$ 29,680.90/year. Therefore, it is estimated that the hospital would save US$ 252.70/month and US$ 3,032.10/year if salinization was always adopted.

## Discussion

The first step of CVC-TI heparinization, “collection of material in the MAC at the 11th floor”, was mainly performed by nursing technicians (96%). This result is consistent with the rational allocation of available resources, since the participation of nurses should be directed to more complex activities, for their professional training, as well as the cost of their direct workforce that is higher than that of nursing technicians.

The time spent in this step had a large variation and the greater expenditure of time was possibly associated with the fact that the MAC at the 11th floor supplies materials for the own floor (Chemotherapy Ambulatory) and for the 12th floor, where the Day Hospital is located. Therefore besides the time spent for locomotion, there was also the time professionals had to wait to collect the material, which depended on the amount of requests and movement in the MAC that operates according to an individualized dispensing system.

Regardless of the professional category, the location of the MAC on the same floor of Day Hospital would reduce the time of travel for collection of supplies used in this and other procedures. However, the different deliberative instances would need to evaluate the intervening variables, among them the viability of the physical structure and the reallocation and/or amplification of the amount of human resources. Although the direct cost of hiring nursing technicians is lower, taking into account their qualification to work in mid-level jobs, especially in highly specialized areas such as cancer, another feasible option would be to investigate the possibility of having other professionals assigned to develop this activity, with a compatible level of qualification.

Specifically with respect to CVC-TI heparinization, the MAC dispensed a 5ml ampule of 100IU/ml heparin of which only 3ml were used and the remaining 2ml discarded. In the Institute, with the individualized dispensing system, the 5ml heparin ampoule was billed per patient, and since patients used each ampoule once only, there was no possibility of packaging or reuse by another patient, thus creating an avoidable waste. From the average of 280.75 procedures performed per month at the Day Hospital, it is estimated that the financial loss related to this type of waste would correspond to US$79.73/month and US$956.80/year.

The occurrence of cancer is associated with economic losses that are difficult to estimate, and the associated financial costs pose a great challenge to universal access health systems, as is the case in Brazil. Thus, care for cancer patients incur many costs and needs to be made feasible in a context of increasing need for investments, finite resources and mandatory search for more effective and efficient strategies^8^. In this perspective, nurses play an important role in different hospital contexts, as they participate in the process of measurement, control and minimization of care costs, contributing to the rational allocation of inputs.

In 2014, a systematic review identified savings in the expenses related to drugs and a decreased occurrence of medication errors by comparing the use of the traditional distribution system with the use of a unitary distribution system. Articles have reported an economy of 14.4% to 67.7% in drug consumption and a reduction of 11.7% to 57% in drug administration errors. It was also pointed out that secondary and tertiary health care institutions use a greater quantity of drugs and other supplies, and thus the use of a unit dose drug distribution system at these levels could represent a greater opportunity for savings^9^.

In the second step, “material preparation and asepsis”, the cost with material was significantly higher in relation to direct cost with the nurse workforce, solutions and additional material, having a strong impact on the total average direct cost of this step and of the complete procedure. The items that most contributed to this outcome were Huber needles, sterile gloves, disposable sterile field and gauze, having been verified the rational consumption of these materials.

Despite recurrent searches in the literature, no studies were found on the cost of CVC-IT heparinization or the cost of maintaining the permeability of similar devices. However, studies^10^ ^-^ ^12^ ^)^ have also highlighted the influence of cost with materials in the calculation of the direct costs of procedures performed by health professionals, especially nursing professionals.

In health institutions, especially public institutions, the scarcity, lack and/or poor quality of consumable goods are observed, causing stress among the multiprofessional team, discontinuation of care and risks of harm to patients. This points to the inexistence of effective and coherent planning in the processes of purchase, control and information on the management of materials^13^.

Technological advances have boosted the increase in care complexity, requiring a high level of specialized attention and creating increasing demands in terms of material resources. In this regard, health services need to improve the managing systems of these resources, providing them in proper quantity and quality to ensure continuity of care at a lower cost. Health professionals, especially nurses, have been pressured to gain knowledge about this issue, to intervene in the management and control of scarce and finite resources, thus adding value to the profession and care^14^.

The third and fourth steps of the procedure, namely, “puncture and CVC-TI heparinization” and “disposal of material”, showed lower average total direct costs than the previous steps and was determined by the cost with the nurse workforce.

We reiterate the need for calculating the direct costs incurred by procedures carried out by nursing professionals, as well as providing an analysis of the consumption of resources to their feasibility and financial impact, so as to provide information to enable the adjustment of supplies and indicate possibilities for improvement in their implementation^4^.

In different work processes, nurses take on the task of supervising the teams under their responsibility, engaging in educational interventions with a view to professional development, and providing direct care to patients in accordance with institutional guidelines. Among these assignments, it is important to note the indispensability of their participation in raising awareness among professionals about the rationalization of expenditures, aiming at minimal waste of resources and commitment with the assistance provided^15^.

It was observed in this study, that the use of some materials in larger quantities than the stipulated, or non-supply of some material (additional material) in the clinical protocol of CVC-IT heparinization, even though without significant costs, indicates the opportunity to sensitize nurses to prevent/minimize waste.

Maintenance of the patency of CVC-IT, as previously mentioned, can be done by replacing heparin with sodium chloride 0.9%. Both have the same purpose and effectiveness, but sodium chloride 0.9% has the advantage of being safer, without anticoagulant-related risks^1^ ^-^ ^2^. A systematic review in 2015 showed that the concentration of heparin alone is not associated with the improvement of central venous catheter patency rates. Furthermore, its systemic effects may cause a problem as they contribute to the development of thrombocytopenia. In contrast, the use of sodium chloride 0.9% has been considered adequate, without significant differences in effectiveness when compared with heparin, and posing a lower risk of complications to the patients^16^.

Therefore, in addition to the scientific evidence available in the literature regarding the benefits of using 0.9% sodium chloride for CVC-IT patency maintenance, the results of the present study provide evidence of economic advantages of using saline solution in the place of heparin. It brings the possibility of cost savings without compromising the quality of the procedure and patient safety.

Finally, we agree that because of their high complexity, health institutions need to adopt economic, managerial and financial instruments to its administration, seeking to offer better budget control and generate gains in terms of efficiency and effectiveness. The use of cost per procedure is a useful method to establish the average price of each health procedure, estimating costs for trading of packages, as well as to contribute to the determination of profitability by stimulating cost control and reduction^17^.

## Conclusion

The average direct total cost of CVC-TI heparinization was US$9.71 (SD=1.35), ranging from US$7.98 to US$23.28, with a median and mode of US$9.51.

Since scientific data supports the use of 0.9% sodium chloride for maintenance of CVC-TI patency, the simulation of its use in place of heparin indicated that no additional material would have to be included in the procedure, and the withdrawal of 20 ml syringes and a 0.9% sodium chloride ampoules would have an average direct total cost of US$8.81 (SD=1.29), achieving a reduction of US$0.90/procedure by the use of salinization.

Although the benefits of 0.9% sodium chloride have been identified for the maintenance of CVC-TI patency, the use of heparin is still common in several health organizations. Therefore, it is expected that the economic outcomes may represent a consistent evidence for nurses, in their spheres of governability, to subsidize their arguments to change the clinical practice, aiming at achieving better patient outcomes and the efficient resource allocation.
